# A New Sign Language for Deaf Mutes

**Published:** 1851-06

**Authors:** Albert J. Myer


ART. II — *A New Sign Language for Deaf Mutes, being a thesis for the degree of Doctor in Medicine, presented and sustained before the Medical Department of the University of Buffalo, Feb.* 25, 1851. By ALBERT J. MYER. Published in accordance with a vote of the Faculty.

The subject I have chosen has, at least, the merit of singularity. How successful I shall be in treating it, or how many converts my crude ideas, sustained by such reasoning as I can offer, may make, is a matter of question. The theory itself, can be better tested by its practical application, than a criticism of its abstract merits.

I might apologize for what may seem an eccentricity, but no one fully realizing my position, can bring that charge. I have thought on other questions; have pondered much on the depth of this, or the beauty of that; but wherever I turned, I found the ground already occupied by the profound treatise of some learned Professor. A mere compilation of notes, or rehearsal of the views of my instructors, seemed hardly to furnish the document called for. My peculiar opportunities for observation have led me to my theme. For hours I have sat at a Telegraph station, watching the strange signs, and listening to the mysterious noises by which muscular action, though five hundred miles distant, was still the medium of influence of mind on mind. I have seen and heard every mental emotion, from the deepest sorrow to the wildest joy, the oppressed movement of wonder, the rapid note of question, the steady converse, and the happy laugh, all expressed by the point and pause of sound, and the very radical of written language—the simple line and dot. It is not strange, therefore, that, under such circumstances, I should not conceive the idea of aiding, with so simple a speech as can be founded on this principle, those whom the Deity has seen fit to deprive of the natural organ.

A language, written by the line and dot, articulated by the long and short sound, to be signaled to the eye by the rise and fall, and to the touch by the interrupted pressure of a finger, appears, even at first thought, peculiarly applicable. Let us see how far material, to sustain this hope, can be gathered from fact, or reasoned from analogy. We turn our attention to those by whom these symbols are oftenest used.

To the thoroughly initiated telegraph operator, his instrument not only writes, but talks. The varying tick, which to you forms no semblance of intelligible sound, may discourse to him most eloquent music. Entice him into conversation, and wrap around his senses every charm of your brilliant sayings, in the midst of your choicest sentence, he shall quietly leave you to answer some question, or to sustain his part upon business that has come to his ear from an hundred leagues. The long reports which fill the columns of our papers, have been taken, and messages are every day transmitted, solely by the measured beating of the armature of an electro-magnet. This is the sign language articulate, and here not theory but practice.

To the ear, the different pauses of sound mark the lines and spaces, and the points mark the dots in their changing arrangement; forming characters as distinct from each other, and as easy recognizable by that organ, as the pronunciation of the letters which they represent. The delicate fibrilloe, which note the difference of vibration between the "I" sound and that of "E," are equally competent to distinguish their articulation upon a scale in which the sound of the first exactly doubles that of the second. How this "reading by sound" may bear on the case of the deaf and dumb, we shall endeavor to show hereafter. But not this alone. He who has perseveringly practiced the system of sign writing we propose, needs no aid from sound, yet may hold converse to his heart's content. What to others may seem the chance movements of a comrade's cane, the idle drumming of a foot, the tapping of a finger as it rests upon the cheek, or, in excess of nerve influence, plays a silent tattoo on the table — to him forms letters, and from letters words, until sentences pass, and thoughts are interchanged, of whose even attempted transference the unskilled might not have the slightest suspicion; and this by characters signaled forth in a *manner*, not only not wonderful, but not even difficult. To the eye, the beat of that finger, as it strikes and as instantly leaves the object on which it moves, and its period of rest as it pauses in contact, form clearly and certainly the dot and line. An organ naturally so sensitive, can require but little tuition to recognize the peculiar movement that indicates their conjunction for each symbol.

The scope of the sign-writer's power should not end here. Once within reach of his comrade, where his hand can but rest on some part of his body, darkness cannot check, nor many thunders silence this voiceless utterance. The eye may be wanting; the ear may be closed; yet the touch shall convey every wish and thought. Here the quick impression, or its longer continuance, distinctly mark the form and location of the only elements of the letters. That the tactile sense, equally with that of sight, or hearing, is capable of perceiving and discriminating between the sensations which indicate different characters, will readily be established by the truths of physiology, or the result of experiment.

Such are the keys to the seeming mystery — a language spoken and understood without the exertion of a solitary organ naturally devoted to the purpose. I have said the performance claims nothing of the marvelous. In fact, it is only strange that so obvious an idea as that of the application of the principle to the uses of mutes, has not long before led to its proposition.

Let any of you, gentlemen, now sitting in judgment on this production, but remember that, by this theory, letters consist solely of lines and dots — let him recall that the long sound to the ear, the long pause to the eye, the long pressure to the touch represent the line, and a point of either the dot, and then decide for himself (by trial if he will) what space would elapse before, either by eye, ear or touch, he could distinguish between a line— formed by holding the finger a second in contact with any object — followed by three taps of one sixteenth of a second each for as many dots — and a similar line followed by only two. Glaring as the distinction seems, it is one of the least marked of the series. Reference to the accompanying alphabet will confirm this assertion, and, with the above easy clue, indicate the requisite motions for each letter. Its examination will also show how easily the dot and line combinations, if found on trial unsuited, can be varied in form, and made to adapt themselves more perfectly to the proposed purpose.

It will hardly be urged that this method of conversation can not be taught to the deaf and dumb. Persons with the complement of sensation can go far in its practice; but by those who most need it, it can be learned in perfection. In the case of these unfortunates, we have to do with a race in whom nature, as if compensating for their sufferings, cultivates to the highest the very organs we call into play. In the absence of any one special sense, the impressibility of those that must act as its proxies, together with the power of the faculties of perception and concentration ever preternaturally increased, and as preternaturally strengthened. Better proof of this recognized fact can not be cited, than the velocity with which this identical class work through the manœuvers of their present alphabet. A series of motions which convey to us no other idea than that of a remarkably continued contortion, and whose rapidity defies our utmost attention, may assist one of them not only with a sentence of beauty, but seem slowly made. Of the "tactus eruditus," our books tell us much, and often are we enjoined to acquire it; but we realize that phrase's fullest meaning, when the erudite finger of the blind student finds pages of printed intelligence in what, to us, is only an incomprehensible roughened sheet of paper.

The eye, whose mere glance can understandingly follow the above mentioned motion, or the touch, possibly trained to such exquisite delicacy, seems little capable of failing in the perception of characters so palpable as those we now offer. With the skill acquired by the practice of years in the constant use of these signals, it would need, for their formation, or the comprehension of their meaning, (when made by others,) hardly a greater amount of volition than we ordinarily exercise in producing, or in recognizing written characters. Without an example, it is difficult to imagine how rapidly a person, forced to express every idea by such media, would convey them to one taught to receive by a similar necessity. The utmost rate to which the requisite motions could be driven, would not baffle senses so accustomed and acute as their's must become. Yet these movements can be made with such velocity, that it tries the pen of a ready writer to note down each letter as quickly as formed. Means for more rapid communication can scarcely be hoped for or desired.

To this speed, we have asserted before, and claim now, as a peerless advantage, darkness could offer no hindrance. Once in contact, the parties converse, by touch, with as perfect a fluency at midnight as at noon-day; — a point, I believe, which no other plan has proposed to accomplish. By that at present in use among mutes, each, in the absence of light, must trace out, finger by finger, the combination which the other forms; and the fact that, however slowly, they can do even this, is a species of evidence how easily and well they might learn this new mode.

We will not here enter into a recital of the many advantages accruing to those for whose benefit we write, from the power alone of being able to converse by signs that need not be seen. They will readily suggest themselves to those who devote either time or attention to the consideration of the subject. For instance; by the method of touch, should one already deaf and dumb, become blind, he could still join his fellows with equal capacity, for all ordinary purposes of conversation, as prior to the loss of his vision. By touch, the blind themselves could readily speak with the deaf and dumb. To those whose joys are so few, even such an extension of acquaintance is a matter of consequence.

Another claim, and which in itself, ought, we think, to decide the matter of preference, is the superiority of this system of signs, whether as addressed to the eye or the touch, in grace and beauty. . The deaf mute might almost cease to be an object of pity. The writhing of fingers, and the anxious effort of sign-making, which now at once challenge our attention, and call for our sympathy, would give place to the quiet, easy movements of the fingers, or, — where perfect secresy was desired between the parties,—

to what would seem perfect repose. We might be long in their company, and they fairly loquacious; while we, in our ignorance, would wonder solely at their reserve and silence. They could sit at our sides — they could walk in our streets, or move in our assemblies, constantly talking, yet attract no other attention, than perhaps the passing remark,— "They must *think* a great deal, for they say very little."

But the dumb shall speak! A startling announcement, but why not true? Because hitherto it has been out of their power to form intelligible sound, must it necessarily remain so? If, to the operator, his *magnet* talks, why may not also the man? The same movement that forms the letter, acting on a proper substance, makes the *sound* of that letter. The tap and pause of the finger that shows "A" to the eye, causes the short and long sound that indicates "A" to the ear. Extend the principle to words, and the speech of the dumb is effected.

There is reason also, why this sign language should be readily acquired. The facility with which the characters of any language are read, or their forms retained, depends on their simplicity. In this point, those we now propose, are reduced to the ultimate,— being formed from the very elements, and farther we can not go. Our final claim, then, is for ready acquisition; and here, for the present, we rest our plea. We have asked much: with what show of justice you must decide. The treatise is not intended so much to demonstrate the results, as to elucidate the propositions, of a theory. Once recognizing the fact, that lines and dots can represent all letters, the modes of indicating them, in their turn, can be multiplied to infinity. The application of this idea to the production of a deaf and dumb alphabet, is all I seek to accomplish.

I do not think myself over enthusiastic. The plan is at least plausible. Older heads than mine have deemed it possible. When every day's press brings its story of the practice, so far carried out, that even the shrieks of a steam-whistle have conveyed information, it surely is not chimerical to imagine that similar principles might be made available in the conversation of rational beings.

The half has not been said, that might be urged, to favor the adoption, or in support of the theory. Physiological details, shewing how and why each sense and each muscle, can acquire the requisite discipline, or instances of its actual possession, in degrees which foreshadow the results to be hoped for from practice, might be given in quantities sufficient to fill a volume. Where there is opportunity for practical experiment, so simple and convincing as that here proffered I have regarded these as hardly more necessary for the purposes of this article, than the descriptive anatomy of every part to be used in the language.

In closing, I have only to ask, from any who may feel disposed hastily to pronounce the proposition impracticable, the simple justice of a fair trial. Let the attention of those, whose experience and study have fitted them to decide on such subjects, be devoted to this but long enough thoroughly to test it, and I shall hope for its speedy and perfect success. We live at an era of changes. The present century has already witnessed stranger things than an audible speech from the deaf and dumb.

The following alphabet is annexed, to afford opportunity for reference or experiment, and possibly better explains the plan at a glance, than can be done by a host of words. The style of letters here used, is that adopted by the Bain telegraph lines. As here printed, the telegraphic signal accompanies the letter which it represents.

### BAIN'S ALPHABET.

| A | B | C | D | E | F | G | H | I | J |
|---|---|---|---|---|---|---|---|---|---|
| - — | - — - | - - - | - - — | - | - — — — | - — - - | - — — | - - | - - - — |

| K | L | M | N | O | P | Q | R |
|---|---|---|---|---|---|---|---|
| - — — - | - - - - | - — - — | - - — — | — | — - — | — - — - | — — - |

| S | T | U | V | W | X | Y | Z |
|---|---|---|---|---|---|---|---|
| — - - | — - - - | — - | — — — - | — — - - | — - - — | — — | — — — |

| & | . | ? | ! | 1 |
|---|---|---|---|---|
| — — — — | - - — - - | — - - — - | — — — — — - | - — — — — |

| 2 | 3 | 4 | 5 | 6 | 7 |
|---|---|---|---|---|---|
| - - — — — | - - - — — | - - - - — | - - - - - | — — — — - | — — — - - |

| 8 | 9 | 0 |
|---|---|---|
| — — - - - | — - - - - | — — - — — |

It may assist in forming the characters, to bear in mind, that whenever the finger *rests* upon the table it is supposed to be drawing a line. Thus, if it touches but for an instant, it makes but a point of such line — a dot.

To write for the EYE, the finger or whole hand, as preferred, should take what we will call a "standard position," at a distance, for instance, of an inch from the table, or other object. From this position, it must always start at the beginning, and to it return at the close, of each letter.

Thus placed, to form a *dot*, bring the hand in contact with the object, and instantly return it to the standard position. For the sake of convenience, we style this "first movement." To form a *line*, bring it down similarly,— retain for a longer period in contact, and in like manner return to place. This is "second movement," and these two are *all*. Perform them in close succession, and you have a dot and a line, ( - —) the first letter of the alphabet, A.

2-0

B (- — -) is precisely similar, with the addition of the "first movement" for the final dot. C (- - -) the "first movement" thrice repeated, while Z (— — —) is the "second movement," repeated in like manner; and so through the list.

The same movements brought to bear upon the hand of a companion, or upon any portion of his body, communicate the sensations necessary for *reading by touch.* If acting on a sonorous substance, they will evidently produce the different sound, or combination of sounds, for each letter, as before mentioned. For all ordinary purposes, the slight tapping, caused by the beating of a finger on the table, will be sufficient.

For the sake of distinctness, a slight interval should be allowed to elapse after forming each letter, before commencing the next. Words are separated by a longer interval.

When the symbols are used for the transmission of "news" by telegraph, words are written almost entirely by abbreviations, as "*fr*" for "from," "*h*" for "have," "*bn*," for "been," &c., &c. This practice would be applicable to the present mode. In this case, the speed would be increased in proportion to the number of such contractions used.

